# Novel BAP degradation pathway during adventitious caulogenesis in *Pinus pinea* L. cotyledons

**DOI:** 10.1186/1753-6561-5-S7-P143

**Published:** 2011-09-13

**Authors:** Ondrej Novak, Candela Cuesta, Karel Dolezal, Lucie Szucova, Lukas Spichal, Belen Fernandez, Ana Rodriguez, Miroslav Strnad

**Affiliations:** 1 Laboratory of Growth Regulators, Faculty of Science, Palacky University & Institute of Experimental Botany AS CR, Slechtitelu 11, Olomouc, CZ-783 71, Czech Republic; 2 Area de Fisiologia Vegetal, Departamento Biologia de Organismos y Sistemas, Instituto de Biotecnologia de Asturias, Universidad de Oviedo, Oviedo, E-33071, Spain

## Background

Cytokinins (CKs) are a group of phytohormones which probably regulate the growth, development, and metabolism of all plants. The aromatic CK benzyladenine (BA) has been widely applied in *in vitro* culture for inducing shoot organogenesis. Our study of endogenous cytokinin profiles during the caulogenic process based on mature cotyledons of stone pine (*Pinus pinea* L.) showed a novel metabolic pathway of aromatic cytokinins based on modification of purine skeleton.

## Methods

Three-year-old mature seeds from two half-sibling selected families and open-pollinated trees of *P. pinea* were used [1] and the samples were collected following the Alonso *et al.*[*2*] procedure. Extraction and purification of cytokinins was based on the method described by Novák et al. [3], including modifications published later [4]. The samples were purified using a combination of a cation (SCX-cartridge) and anion [DEAE-Sephadex/C18-cartridge] exchangers. Combination of high performance liquid chromatography (HPLC) with quadrupole-time of flight mass spectrometry (QqTOF) was used for accurate and sensitive identification and quantification of cytokinins.

## Results and discussion

Cortizo et al. [5] published the dynamics of BA uptake and metabolism in *P. pinea* cotyledons excised from embryos precultured for 2 and 4 days and cultured *in vitro* in modified Le Poivre media with 4.4 µM BA. In our experiment, we used 44.4 µM concentration of BA and samples were collected at different periods (0; 1; 2; 6; 16; 24 h and 2; 4; 6 d). Using high-resolution MS, the naturally-occurring BA metabolites as well as new BA forms were identified. In comparison with previously published profiles of the BA metabolite pool [5,6], the ribosyl and glycosyl forms were quantified as the most abundant metabolites. Moreover the biological activity of identified BA and its derivatives were compared in various CK bioassays. The results indicate that BA uptake during the caulogenic process may be possible to regulate not only by known cytokinin pathways. Finally, the feeding experiment with stable isotope-labelled standard, ^15^N_4_-BA, confirmed our identification of the novel metabolic CK pathway.

## Conclusions

The identification of the novel BA forms demonstrates that the novel cytokinin pathway is used as a control mechanism of BA uptake from the bud induction medium during adventitious caulogenesis in cotyledons of *P. pinea*. Our results help to understand the processes associated with embryo germination in plant tissue culture.
